# *Achromobacter* Species: An Emerging Cause of Community-Onset Bloodstream Infections

**DOI:** 10.3390/microorganisms10071449

**Published:** 2022-07-18

**Authors:** Burcu Isler, David L. Paterson, Patrick N. A. Harris, Weiping Ling, Felicity Edwards, Claire M. Rickard, Timothy J. Kidd, Ian Gassiep, Kevin B. Laupland

**Affiliations:** 1 UQ Centre for Clinical Research, Faculty of Medicine, University of Queensland, RBWH Campus, Brisbane 4029, Australia; burcu.isler@uq.edu.au (B.I.); padstock@hotmail.com (P.N.A.H.); w.ling@uq.edu.au (W.L.); c.rickard@uq.edu.au (C.M.R.); i.gassiep@uq.edu.au (I.G.); 2 Infection Management Services, Princess Alexandra Hospital, Brisbane 4102, Australia; 3 Royal Brisbane and Women’s Hospital, Brisbane 4029, Australia; k.laupland@qut.edu.au; 4 Central Microbiology Laboratory, Pathology Queensland, Brisbane 4006, Australia; timothy.kidd@health.qld.gov.au; 5 Faculty of Health, School of Clinical Sciences, Queensland University of Technology, Brisbane 4072, Australia; f.edwards@qut.edu.au; 6 Herston Infectious Diseases Institute to the Metro North Health, Metro North Health, Brisbane 4029, Australia; 7 School of Chemistry and Molecular Biosciences, The University of Queensland, Brisbane 4072, Australia

**Keywords:** *Achromobacter*, bloodstream infection, incidence

## Abstract

Background: Case reports and small series indicate that *Achromobacter* species bloodstream infection (BSI) is most commonly a complication of hospitalization among patients with chronic lung disease. The aim of the present study was to determine the incidence, risk factors, and outcomes of *Achromobacter* sp. BSI in an Australian population. Methods: Retrospective, laboratory-based surveillance was conducted in Queensland, Australia (population ≈ 5 million) during 2000–2019. Clinical and outcome data were obtained by linkage to state hospital admissions and vital statistics databases. BSI diagnosed within the community or within the first two calendar days of stay in hospital were classified as community-onset. Community-onset BSIs were grouped into community-associated and healthcare-associated. Results: During more than 86 million person-years of surveillance, 210 incidents of *Achromobacter* sp. BSI occurred among 195 individuals for an overall age-and sex-standardized annual incidence of 2.6 per million residents. Older individuals and males were at highest risk (2.9 vs. 2.0 per million, IRR for males 1.5; 95% CI, 1.1–1.9; *p* = 0.008). Most (153; 73%) cases were of community-onset of which 100 (48%) and 53 (25%) were healthcare- and community-associated, respectively. An increasing proportion of community-onset cases were observed during twenty years of surveillance. Underlying medical illnesses were common with median (interquartile range) Charlson Comorbidity Index (CCI) scores of 3 (1–5). CCI scores of 0, 1, 2, and 3+ were observed in 37 (18%), 27 (13%), 40 (19%), and 105 (50%) of cases, respectively. All but one of the cases were admitted to hospital for a median (interquartile range) length of stay of 12 (5–34) days. All-cause case–fatality rates in hospital by day 30 and by day 90 were 30 (14%), 28 (13%), and 42 (20%), respectively. The 90-day case–fatality rate increased with increasing comorbidity and was 3% (1/37), 11% (3/27), 25% (10/40), and 27% (28/105) among those with Charlson Comorbidity Indices of 0, 1, 2, and 3+, respectively (*p* = 0.004). Conclusions: Although comorbidity is an important determinant of risk, most *Achromobacter* sp. BSI are of community-onset and one-fifth of cases occur in patients without significant underlying chronic co-morbidities. This study highlights the value of population-based methodologies to define the epidemiology of an infectious disease.

## 1. Introduction

*Achromobacter* is a genus of non-fermentative Gram-negative bacillus, which is primarily found in respiratory tract specimens from patients with cystic fibrosis. However, the genus is also recognized to be an emerging group of pathogens that have been recognized to primarily cause hospital-acquired infections among patients with significant comorbid illness [1]. They are environmental, low-virulence organisms with infections rarely reported to occur in otherwise healthy hosts. These bacteria have a propensity to cause infection by forming biofilms. Antimicrobial therapy is challenged by this fact, along with other intrinsic and acquired resistance mechanisms [2,3,4]. While lung infection is the most commonly recognized clinical syndrome, invasive infections including meningitis, endocarditis, and bloodstream infection (BSI) may occur. In one case series and review of 77 cases reported in the literature, *Achromobacter xylosoxidans* BSI was found to have a case–fatality rate of 30% [5]. Treatment is complicated by intrinsic resistance to aminoglycosides, aztreonam and most cephalosporins. Acquired resistance to a variety of antimicrobials is also increasingly commonplace [4].

Knowledge on the epidemiology of *Achromobacter* species BSI is limited to small case reports and series from selected populations [4,6,7]. These types of studies are at significant risk of a number of biases that may lead to inaccurate conclusions surrounding the epidemiology of an infectious disease. Therefore, this study aimed to define the incidence, risk factors, and outcome of *Achromobacter* sp. BSI in a large population-based Australian cohort to establish the burden of illness [8].

## 2. Methods 

### 2.1. Study Population

All Queensland residents with *Achromobacter* BSI within the publicly funded system between 1 February 2000 and 31 December 2019 were identified and included in the study. Pathology Queensland is the largest pathology laboratory in Queensland and is publicly funded. Private healthcare services were not included [9].

### 2.2. Microbiologic Methods

The BACT/ALERT^®^ 3D system (bioMérieux, Durham, NC, USA) was used until 2018. BACT/ALERT^®^ VIRTUO^®^ system (bioMérieux, Durham, NC, USA) was used from 2018.

BacT/ALERT FA plus (aerobic), FN plus (anaerobic), and PF plus (paediatric) media bottles were used for culture. Incubation period was 5 days before discarding for no growth.

Species identification was performed using VITEK^®^ GN ID, API 20NE and MALDI-TOF MS. Antibiotic susceptibility testing was performed using VITEK^®^ AST card, and disc diffusion as per CLSI or EUCAST at the time of testing.

All blood cultures with growth of *Achromobacter* sp. were retrospectively identified by the Clinical Information Systems Support Unit, Queensland Health. Incident BSI was the first isolation of a *Achromobacter* sp. per patient. All subsequent isolations of the same species within 30 days were considered as part of the same episode. Polymicrobial infection was defined as the BSI where another significant pathogen was isolated from the blood culture in addition to *Achromobacter* sp. within a 48 h period [10].

### 2.3. Data Linkage

Clinical and outcome information was obtained through linkages to statewide databases. A linkage was performed with the Queensland Hospital Admitted Patient Data Collection (QHAPDC) to obtain all healthcare encounters associated with private and public institutions, covering the time period one year before and one year after the index blood culture.

All healthcare encounters associated with private and public institutions were obtained through a linkage with the Queensland Hospital Admitted Patient Data Collection (QHAPDC). Other important variables including hospital admission and discharge dates, diagnostic codes and discharge survival status were obtained through QHAPDC. Multiple admission episodes within a continuous period (such as with inter-hospital transfers) were considered to belong to a single hospital admission for the purposes of length of stay. Deaths were confirmed through The Registry of General Deaths in Queensland.

### 2.4. Definitions

BSI diagnosed within the community or within the first two calendar days of stay in hospital were classified as community-onset (CO). Community-onset BSIs were grouped into two, as community-associated (CA) and healthcare-associated (HCA). Healthcare-associated BSIs were those community-onset BSIs that occurred among nursing home residents, and those who had encounters at a healthcare institution within 30 days and/or admission to hospital for more than two days within the 90 days prior to index blood culture [11]. Community-onset BSIs that did not fulfill criteria for healthcare-associated infections were classified as community-associated.

BSIs were classified as hospital-onset (HO) if the index blood culture was drawn two calendar days after admission or within two calendar days of hospital discharge [11]. Co-morbid medical illnesses were defined using the Charlson Comorbidity Index [12,13]. The Charlson Comorbidity Index is a method of predicting mortality by classifying or weighting comorbid conditions. A clinical focus was assigned based on review of diagnosis-related group and primary diagnosis hospital discharge codes.

### 2.5. Statistics

Data were analysed using Stata 16.1 (StataCorp, College Station, TX, USA). The primary units of analysis were incident BSI episodes and were reported as age- and sex-standardized (to 2019 Queensland population) annual rates per million population. Non-residents of Queensland were excluded. Denominator data were stratified by age and sex. The hospital and health service area was obtained from Queensland Health using data available from the Australian Bureau of Statistics [14]. The total annual number of sets of blood cultures performed by Pathology Queensland was obtained [15]. Prior to analysis of continuous data, the underlying distribution was assessed using histograms. Medians with interquartile ranges (IQR) were used to describe skewed variables and groups were compared using the Mann–Whitney–Wilcoxon test. Categorical variables were reported as proportions and compared using Fisher’s exact test. Incidence rate ratios (IRR) with exact 95% confidence intervals (CI) were calculated for group comparison. A two-tailed *p*-value < 0.05 was considered to represent statistical significance.

### 2.6. Ethical Approval

Ethics approval was obtained from the Royal Brisbane and Women’s Hospital’s human research ethics committee with a waiver of individual consent (LNR/2020/QRBW/62494).

## 3. Results

During 86 million person-years of surveillance, 210 incident *Achromobacter* sp. BSI occurred among 195 individuals resulting in an annualized age and sex-standardized incidence of 2.6 per million residents. Ten people had second, three had third, and two had a fourth episode(s) of incident *Achromobacter* sp. BSI. Among the 210 incident cases, 57 (27%) were classified as hospital-onset, 100 (48%) as healthcare-associated, and 53 (25%) as community-associated BSIs.

### 3.1. Incidence

**Figure 1:**
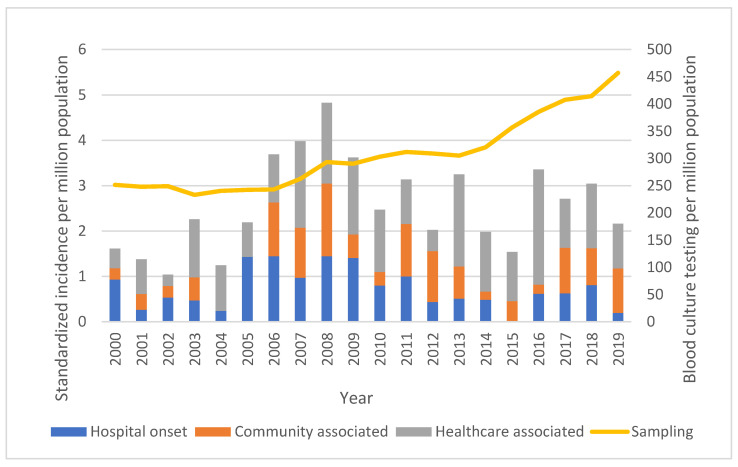
Age- and sex-standardized incidence of *Achromobacter* sp. BSI in Queensland.

**Figure 2:**
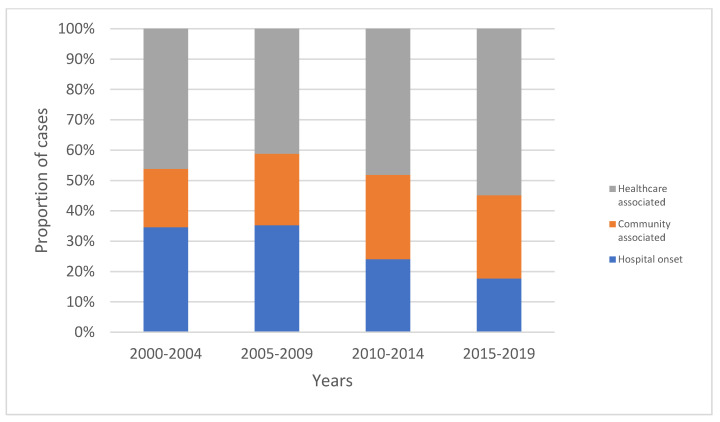
Proportions of healthcare-associated, community-associated and hospital-onset BSI due to *Achromobacter* species between 2000 and 2019.

Although there was substantial year to year variability, no trend for a change in the overall age- and sex-standardized incidence of *Achromobacter* sp. BSI was observed, as shown in Figure 1. However, there was a significant increasing proportion of cases that were community-onset, with increases in both community-associated and healthcare-associated BSIs, as shown in Figure 2 (*p* = 0.011).

### 3.2. Demographic Risk Factors for Acquisition

**Figure 3:**
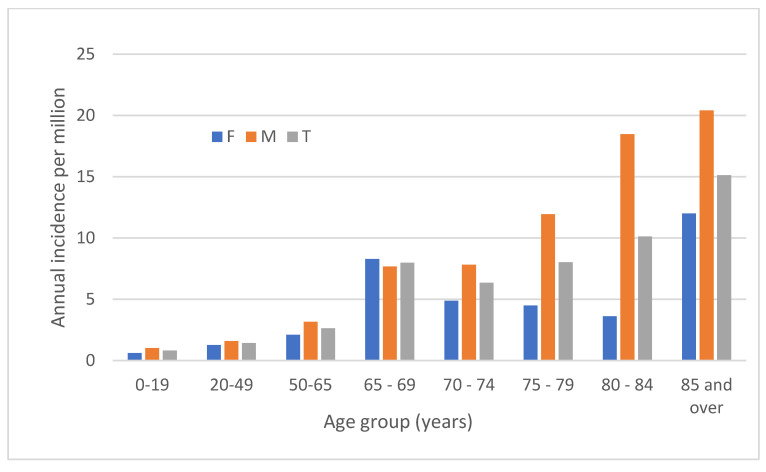
Age and sex of BSI cases. F, female; M, male; T, total.

The median age was 61.0 (IQR, 41.5–72.1) years and 124 (59%) incident episodes were in males. The incidence of BSI by sex and age is shown in Figure 3. Although the excess risk varied considerably by age, overall males were at significantly higher risk for developing *Achromobacter* sp. BSI (2.9 vs. 2.0 per million, IRR for males 1.5; 95% CI, 1.1–1.9; *p* = 0.008).

**Figure 4:**
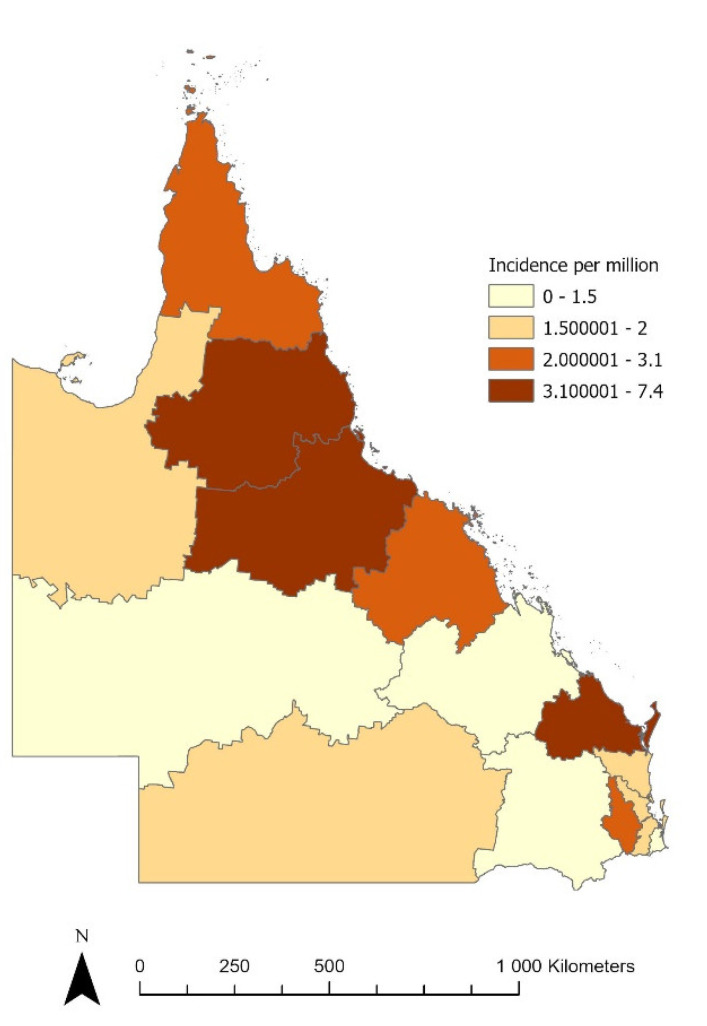
The incidence of *Achromobacter* sp. by geographic location.

The incidence of *Achromobacter* sp. BSI demonstrated significant variation by hospital and health service area, with the highest rates observed in the coastal areas north of latitude 25° S (annual incidence of 5.6 per million residents vs. 1.69 per million residents elsewhere in the state; incidence rate ratio (IRR) 3.32; 95% confidence interval 2.50–4.39; *p* < 0.0001), as shown in Figure 4. However, no significant (*p* = 0.6) relationship between region and onset classification (HO, CA, or HA) was observed.

### 3.3. Clinical Determinants

Underlying medical illnesses were common among individuals with *Achromobacter* sp. BSI with median (IQR) Charlson Comorbidity Index scores of 3 (1–5). Scores of zero, 1, 2, and 3+ were observed in 37 (18%), 27 (13%), 40 (19%), and 105 (50%) of cases, respectively.

The most common Charlson co-morbidities observed included renal disease, diabetes mellitus, and malignancies, as shown in Table 1. Among those with co-morbidities, 40 haemodialysis patients, three patients with cystic fibrosis, and five organ/tissue transplant recipients were identified. Among the 38 patients with no Charlson Comorbidity identified, illicit drug use was identified in two patients.

The most common clinical foci were endovascular and soft tissue infections, and these varied among cases according to the presence of co-morbidities as shown in Table 1. Among the endovascular infections, 19 were coded as infected cardiac/vascular device/implant/grafts, two as unspecified arteritis, and one each as endocarditis and thrombophlebitis. Fewer than 10% of patients had a respiratory tract focus of infection associated with their bacteraemia (Table 1).

### 3.4. Antimicrobial Susceptibility

Antimicrobial resistance testing results showed isolates that were susceptible, intermediate, and resistant to ciprofloxacin (*n* = 198) in 72 (36%), 7 (4%), and 119 (60%) cases; they were susceptible, intermediate, and resistant to gentamicin (*n* = 203) in 21 (10%), 0, and 182 (90%) cases; they were susceptible, intermediate, and resistant to meropenem (*n* = 182) in 178 (98%), 0, and 4 (2%) cases; they were susceptible, intermediate, and resistant and co-trimoxazole (*n* = 190) in 176 (93%), 0, and 14 (7%) cases, respectively (Table 2).

### 3.5. Hospital Admission and Outcome

All but one of the cases were admitted to hospital for a median (interquartile range) length of stay of 12 (5–34) days. Thirty, 28, and 42 patients died in-hospital, by day-30, and by day-90 for all-cause case–fatality rates of 14%, 13%, and 20%, respectively. The 90-day case–fatality rate increased with increasing comorbidity and was 3% (1/37), 11% (3/27), 25% (10/40), and 27% (28/105) among those with Charlson Comorbidity Indices of 0, 1, 2, and 3+, respectively (*p* = 0.004).

## 4. Discussion

In this study, we report the incidence, determinants, and outcome of *Achromobacter* sp. BSI in a large Australian population. This study documents the relatively infrequent occurrence of these infections and suggests that comorbidity, age, and sex may be important determinants for their acquisition. This novel study finds, in contrast to previous findings, that the majority of these infections are of community-onset and have been increasing in number in recent years [4,7]. There is a paucity of data on *Achromobacter* BSIs and much that has been reported is from tertiary care centres. To date, three case series of *Achromobacter* BSI were reported from three different tertiary care centres, one being a cancer centre, with a total number of 113 patients [7,14,15]. All patients had several underlying comorbidities, and infection-related mortality rate was 15–23%, likely reflecting the effect of underlying comorbidities and disease severity. Our report spans tertiary centres specialising in thoracic transplantation and cystic fibrosis through to small rural hospitals.

From an epidemiological viewpoint, we are the first to describe an association of the infection with geographic locations. We found that the incidence was highest in coastal areas north of latitude 25° S (annual incidence of 5.6 per million residents vs. 1.69 per million residents elsewhere in the state). An association between humidity and *Achromobacter* surgical site infection prevalence was previously suggested by others [16]. In the state of Queensland, community acquired infections with other non-fermentative Gram-negative bacilli (*Burkholderia pseudomallei*, *Burkholderia cepacia* complex and *Acinetobacter baumannii*) are also found in these more tropical locations [17]. While we could not find an association between region and community-associated infections, further investigation into environmental reservoirs in more tropical locations is needed.

The relative infrequency of *Achromobacter* BSI occurring in patients with cystic fibrosis or of respiratory tract source is noteworthy. In contrast, disorders where there is frequent use of intravascular access devices (renal disease, which requires haemodialysis and cancer) are logical sources of *Achromobacter* bloodstream infection [18]. This is in keeping with the ubiquity of the organism in this environment, especially in aquatic locations. In our study the overall mortality of *Achromobacter* BSI was 20% by day-90, rising to 27% in patients with 3 or more comorbidities as denoted on the Charlson Comorbidity Index. This is as high as the mortality rates for the BSIs caused by more commonly encountered pathogens, hence would warrant more attention [19].

*Achromobacter* sp. are intrinsically resistant to a variety of antibiotic classes [4]. We did not have the granularity of data to be able to describe treatment choices for each particular infection nor if inadequate choice was associated with higher mortality. Almost two-thirds of our isolates were non-susceptible to fluoroquinolones, further limiting antibiotic choice. Although rare, we also had isolates which were resistant to carbapenems or trimethoprim-sulfamethoxazole. Carbapenem resistance in *Achromobacter* is mainly caused by multidrug efflux pumps and metallo-beta-lactamases [4], and further investigation into mechanisms of resistance in our isolates would be worthwhile. Neither cefiderocol nor eravacycline were commercially available in Queensland at the time of this study, but both antimicrobials have been used with success as salvage therapy in emerging reports [4].

While our study has a number of strengths, there are some limitations that merit discussion. First, this study was retrospective and limited to data already collected in existing databases. As a result, we were not able to tailor data collection to gather details on variables. Perhaps most importantly is the classification of focus of infection and predisposing factors that would have been more accurate with chart review and prospective data collection. Second, our study was limited to cultures performed within the publicly funded healthcare system and cases presenting to private hospitals were not included. While we suspect that this represents a small proportion of cases overall, our incidence rates are therefore conservative and potential underestimates of the true number of cases. Third, like with all studies examining BSI, ascertainment of cases requires that a specimen of blood be submitted and subsequently cultured positive. There were no specific protocols directing physicians to order these tests and the possibility exists that bias could have been introduced in the study if decisions to order blood cultures varied among areas or over time. The increase in testing over the years was not included in the statistical model when evaluating community-onset BSI as there is no accepted “correction factor” that allows adjustment for this. Last, we were not able to determine the precise identity of the organisms here with more sophisticated and gold standard techniques such as MLST/sequence-based identification. As such, the true incidence of each of the more than 15 currently described human disease-causing *Achomobacter* sp. remains unclear.

In summary, this novel study details the epidemiology of *Achromobacter* sp. BSI in a large Australian population over two decades and finds that in contrast to the body of previous literature generated from case reports and small series that *Achromobacter* sp. are predominantly causes of community onset BSI. These data highlight the importance of study designs to minimize selection biases in the establishment of the epidemiology of an infectious disease.

## Figures and Tables

**Table 1:** Clinical characteristics of bloodstream infections due to *Achromobacter* sp. among individuals with and without underlying co-morbid medical illnesses, Queensland, 2000–2019. Mann–Whitney–Wilcoxon test was used to compare the groups. Categorical variables were reported as proportions and compared using Fisher’s exact test.

Variable	No Charlson Comorbidity (*n* = 38)	Charlson 1 or More (*n* = 172)	*p*-Value
Onset classification			0.035
Hospital-onset	9 (24%)	48 (28%)	
Healthcare-associated	13 (34%)	87 (51%)	
Community-associated	16 (42%)	37 (22%)	
Male sex	26 (68%)	98 (57%)	0.2
Charlson variables	-		-
Myocardial infarction		26 (15%)	
Congestive heart failure		45 (26%)	
Peripheral vascular disease		32 (19%)	
Cerebrovascular disease		13 (8%)	
Dementia		4 (2%)	
Chronic pulmonary		39 (23%)	
Rheumatic		5 (3%)	
Peptic ulcer disease		5 (2%)	
Liver disease		26 (15%)	
Diabetes mellitus		62 (36%)	
Plegia		15 (9%)	
Renal disease		82 (48%)	
Malignancy		48 (28%)	
Focus of infection			<0.001
No focus identified	10 (27%)	90 (52%)	
Soft tissue	3 (8%)	20 (12%)	
Bone and joint	5 (14%)	8 (5%)	
Head and neck	3 (8%)	0	
Lower respiratory	2 (5%)	15 (9%)	
Endovascular	7 (19%)	19 (11%)	
Abdominal	3 (8%)	16 (9%)	
Urinary/pelvic	4 (11%)	4 (2%)	
Polymicrobial infection	8 (21%)	39 (23%)	1.0

**Table 2:** Antimicrobial susceptibility testing of *Achromobacter* sp.

Antibiotic Name (Number of Isolates Tested)	Susceptible, *n* (%)	Intermediate, *n* (%)	Resistant, *n* (%)
Ciprofloxacin (198)	72 (36)	7 (4)	119 (60)
Gentamicin (203)	21 (10)	0	182 (90)
Meropenem (182)	178 (98)	0	4 (2)
Co-trimoxazole (190)	176 (93)	0	14 (7)

## Data Availability

Data is available upon request from the corresponding author.
